# Antipsychotic Prescribing Practice in Adults With a First Presentation of Psychosis Amongst Bolton's Early Intervention Team and Inpatient Mental Health Services: An Audit

**DOI:** 10.1192/bjo.2022.469

**Published:** 2022-06-20

**Authors:** Eve Morrison, Hannah Cappleman

**Affiliations:** Early Intervention in Psychosis Team, Bolton, United Kingdom

## Abstract

**Aims:**

In Bolton Early Intervention Team (EIT) it was noticed that patients prescribed antipsychotics frequently required a change in medication due to side effects. Similar issues had been identified in Avon and Wiltshire NHS Foundation Trust where a prescribing guideline was developed which won the NICE Shared Learning Award in 2020. This recommends prescribing Aripiprazole first line and cautions using Olanzapine or typical antipsychotics first due to their side effects. The aim of this project was to identify which antipsychotic drugs are currently prescribed in first episode of psychosis (FEP) in Bolton EIT patients and to audit adherence to National Institute of Clinical Excellence antipsychotic prescribing guideline CG178.

**Methods:**

The sample included all adults with FEP accepted by Bolton EIT across a four-month period from 01/12/20 until 31/03/21. Fifty-two people were identified.

Measured standards were documentation of prescribing rationale, discussion regarding medication side effects and weekly weight monitoring for six weeks following initiation. Antipsychotic choice and need for a change within six months of initiation was recorded. Data were collected retrospectively from patients’ electronic records.

**Results:**

Thirty-eight patients had been prescribed an antipsychotic – fifteen as inpatients, seventeen by Bolton EIT and six by the Home Treatment Team.

Of the fifteen inpatients Olanzapine (8) and Zuclopenthixol (3) were the most common choice. 5/15 had a documented rationale, and side effects were discussed with 3/15 patients. Weekly weight monitoring was performed in 7/15.

Of the 17 people who started antipsychotic medication once under Bolton EIT Quetiapine (6), Olanzapine (6) and Aripiprazole (5) were the most common choices. 12/17 had a documented rationale and 13/17 were consulted regarding side effects. Weekly weight monitoring was not performed for any of these patients.

Within six months, sixteen antipsychotic prescriptions (42%) were changed due to side effects (9), inefficacy (6) and non-compliance (1). The drugs changed were Olanzapine (6) Quetiapine (6) Zuclopenthixol (2) Aripiprazole (1) and Chlorpromazine (1).

**Conclusion:**

Those initiated on antipsychotics as inpatients need better involvement in decision-making and consultation about side effects. A community initiative should be introduced to offer weekly weight monitoring. Further work is required to understand the rationale for frequently prescribing Olanzapine and Zuclopenthixol in inpatient services, and to consider why Aripiprazole is infrequently used first line.

